# Imported *Leptospira licerasiae* Infection in Traveler Returning to Japan from Brazil

**DOI:** 10.3201/eid2303.161262

**Published:** 2017-03

**Authors:** Motoyuki Tsuboi, Nobuo Koizumi, Kayoko Hayakawa, Shuzo Kanagawa, Norio Ohmagari, Yasuyuki Kato

**Affiliations:** National Center for Global Health and Medicine, Tokyo, Japan (M. Tsuboi, K. Hayakawa, S. Kanagawa, N. Ohmagari, Y. Kato);; National Institute of Infectious Diseases, Tokyo (N. Koizumi)

**Keywords:** *Leptospira*, leptospirosis, Brazil, intermediate, *Leptospira licerasiae*, zoonosis

## Abstract

We describe a case of intermediate leptospirosis resulting from *Leptospira licerasiae* infection in a traveler returning to Japan from Brazil. Intermediate leptospirosis should be included in the differential diagnosis for travelers with fever returning from South America. This case highlights the need for strategies that detect pathogenic and intermediate *Leptospira* species.

Leptospirosis, caused by spirochetes of the genus *Leptospira*, is a neglected zoonotic disease found in tropical and subtropical regions. *Leptospira* species are classified into 3 groups on the basis of 16S rRNA gene sequences: pathogenic, intermediate, and saprophytic groups. Although *Leptospira* species from the pathogenic group are considered to be the main cause of leptospirosis, Chiriboga et al. reported that most cases of leptospirosis in Ecuador were caused by intermediate species (*1*). We describe a case of leptospirosis caused by *L. licerasiae*, an intermediate species, in a traveler returning to Japan from Brazil.

In late November 2015, a previously healthy 40-year-old Japanese man sought treatment at the National Center for Global Health and Medicine (Tokyo, Japan) with a high fever and shaking chills. He had recently spent 15 days in Corumbá, Brazil, where he worked as part of a camera crew in mid-November 2015. He used insect repellent during his trip but had been bitten by mosquitoes many times while walking and swimming in waist-deep water in the Brazilian wetlands. His symptoms began 6 days after his return from Brazil and included high fever, chills, arthralgia and myalgia in his elbow and knee joints, and burning skin pain over the his whole body for the 24 hours before he sought treatment. At the time of his first visit to this clinic, he reported new onset retroorbital pain and shaking chills.

On examination, his body temperature was 39.5°C and his pulse rate was relatively low (87 beats/min). He had mild congested bulbar conjunctivae, localized urticaria on his trunk, and many small, old injury scars on both of his legs (Technical Appendix Figure 1). Results of rapid antigen detection tests and Giemsa stains of blood smears for *Plasmodium* spp. were negative for 3 consecutive days. Results of laboratory tests were negative, including IgM, IgG, and NS-1 antigen tests against dengue virus; HIV screening; rapid antigen detection test against influenza virus; and blood and urine cultures. Moreover, PCR results for *Leptospira* and for dengue, chikungunya, and Zika viruses were negative.

Treatment with ceftriaxone (2 g 1×/d) was initiated 1 day after hospital admission. Four hours after infusion began, the patient’s fever rose to 40°C, which was considered a Jarisch-Herxheimer reaction. Fever resolved the next day. Laboratory test results showed elevated total bilirubin (2.3 mg/dL [reference range 0.3–1.2mg/dL]), aspartate aminotransferase (62 U/L [13–33 U/L]), alanine aminotransferase (73 U/L [8–42 U/L]), lactic acid dehydrogenase (456 U/L [119–229]), and C-reactive protein (13.6 mg/dL [0–0.3 mg/dL]), but these values quickly returned to within reference ranges. Three days after ceftriaxone treatment began, all symptoms had resolved, and the patient was discharged from the hospital with a prescription for doxycycline (100 mg 2×/d).

At the time of discharge, 3 days after the blood culture was set up, spirochetes were observed in Korthof and EMJH media. Nucleotide sequencing of the 16S rRNA gene of the isolate, NIID18 (Japan National Bioresource of Bacterial Pathogens no. 18467, http://pathogenic.lab.nig.ac.jp/) (Technical Appendix Figure 2), revealed it to be *L*. *licerasiae*: the sequence (GenBank accession no. LC164227) had 99.3% identity (1,339/1,348 bp) with VAR 010 (GenBank accession no. EF612284), the type strain of *L. licerasiae*. The partial *flaB* sequence of the NIID18 isolate (GenBank accession no. LC164228) also showed the highest similarity with VAR 010 (96.6%, GenBank accession no. LC005426). NIID18 did not react with a panel of antisera for 18 serovars (*2*). An increase in antibody titers in paired serum samples was observed against the isolate (reciprocal titers 50 and 200 in acute- and convalescent-phase samples, respectively), according to microscopic agglutination test (*3*). After receiving antimicrobial drug therapy for 7 days, the patient had completely recovered.

The intermediate *Leptospira* group comprises 5 species: *L. licerasiae*, *L. wolffii*, *L. fainei*, *L. broomii*, and *L. inadai*. Although this species group has been detected in environmental soil and water samples from the Southeast Asia (*4*–*6*), human cases involving returned travelers have not been well-documented previously (*1*,*7*–*10*). To our knowledge, only 2 cases of *L. licerasiae* isolation from a human host have been reported; such isolations were first reported in Peru in 2008 (*7*) (Table), although many serum samples from febrile patients in the Peruvian Amazon have reacted with an *L. licerasiae* isolate. Members of *Rattus* species are considered major reservoir hosts (*7*).

**Table:** Case descriptions of patients infected with *Leptospira licerasiae *in South America*

Patient no.	Age, y/sex	Location	Occupation	Symptoms	Therapy	Prognosis	Ref.
VAR10	31/F	Varillal, Peru	Food vendor	2-d history of fever, malaise, chills, headache, dizziness	Antipyretics	Resolved 5 d later	(*7*)
HAI029	19/F	Iquitos, Peru	Student/ domestic worker	5-d history of fever, malaise, chills, headache, dizziness, leg pain and weakness, abdominal pain, anorexia, nausea, vomiting	None	Resolved†	(*7*)
NIID18	40/M	Corumbá, Brazil	Camera crew	1-d history of fever, shaking chills, retroorbital pain, arthralgia and myalgia in elbow and knee joints, burning skin pain over the whole body, congested bulbar conjunctivae	CTRX 2 g/d for 4 d, then DOX 100 mg 2×/d for 3 d	Resolved within 3 d after initiation of CTRX	This study

*CTRX, ceftriaxone; DOX, doxycycline; ref., reference. †The day when the symptoms resolved was not described in the report.

We were unable to detect *Leptospira* DNA in the case-patient’s blood using *flaB*-nested PCR because this method is specific to species in the pathogenic group. The patient received a diagnosis of leptospirosis after *L. licerasiae* was isolated from a blood culture. Therefore, PCR targeting conserved genes among genus *Leptospira*, such as 16S rRNA, is more suitable not only for clinical situations but also for epidemiologic studies.

This case highlights the need for including leptospirosis caused by intermediate group species in the differential diagnosis for patients with fever who have recently returned from South America. In addition, we emphasize the utility of genes such as 16S rRNA for detecting pathogenic and intermediate *Leptospira* groups.

Technical Appendix Figures showing congested bulbar conjunctivae and skin rash on the trunk of the patient with *Leptospira licerasiae* infection at initial hospital visit and also the biological characteristics of *Leptospira *isolate NIID18 obtained from the patient with *L. licerasiae* infection.
