# Supplementary material for: Imported Leptospira licerasiae Infection in Traveler Returning to Japan from Brazil
Source: Emerg Infect Dis. 2017 Mar;23(3):548–9. doi: 10.3201/eid2303.161262 (PMC5382744; doi:10.3201/eid2303.161262)
Supplement: Technical Appendix — Figures showing congested bulbar conjunctivae and skin rash on the trunk of the patient with Leptospira licerasiae infection at initial hospital visit and also the biological characteristics of Leptospira isolate NIID18 obtained from the patient with L. licerasiae infection. [file 16-1262-Techapp-s1.pdf]

Article DOI: http://dx.doi.org/10.3201/eid2303.161262

Imported *Leptospira licerasiae* Infection in Traveler Returning to Japan from Brazil

Technical Appendix

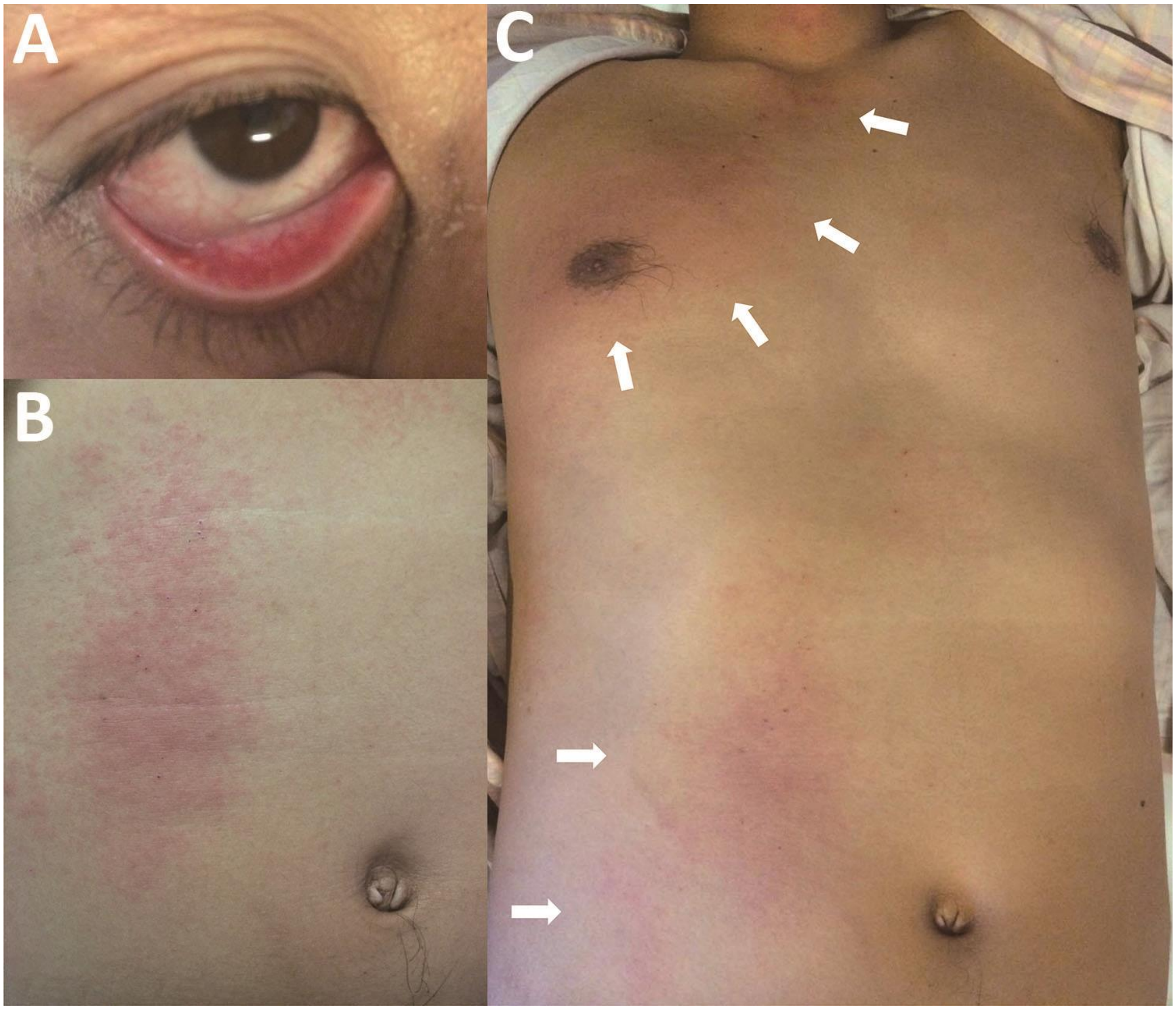

Technical Appendix Figure 1. Congested bulbar conjunctivae and skin rash on the patient's trunk at initial hospital visit of patient with *Leptospira licerasiae* infection. A) Congested bulbar conjunctivae resolved shortly after the initiation of antibiotics. (B, C) Localized skin rash (arrows) on the patient's trunk was treated with antihistamine cream. The relationship between *Leptospira* infection and skin rash was unclear in this case.

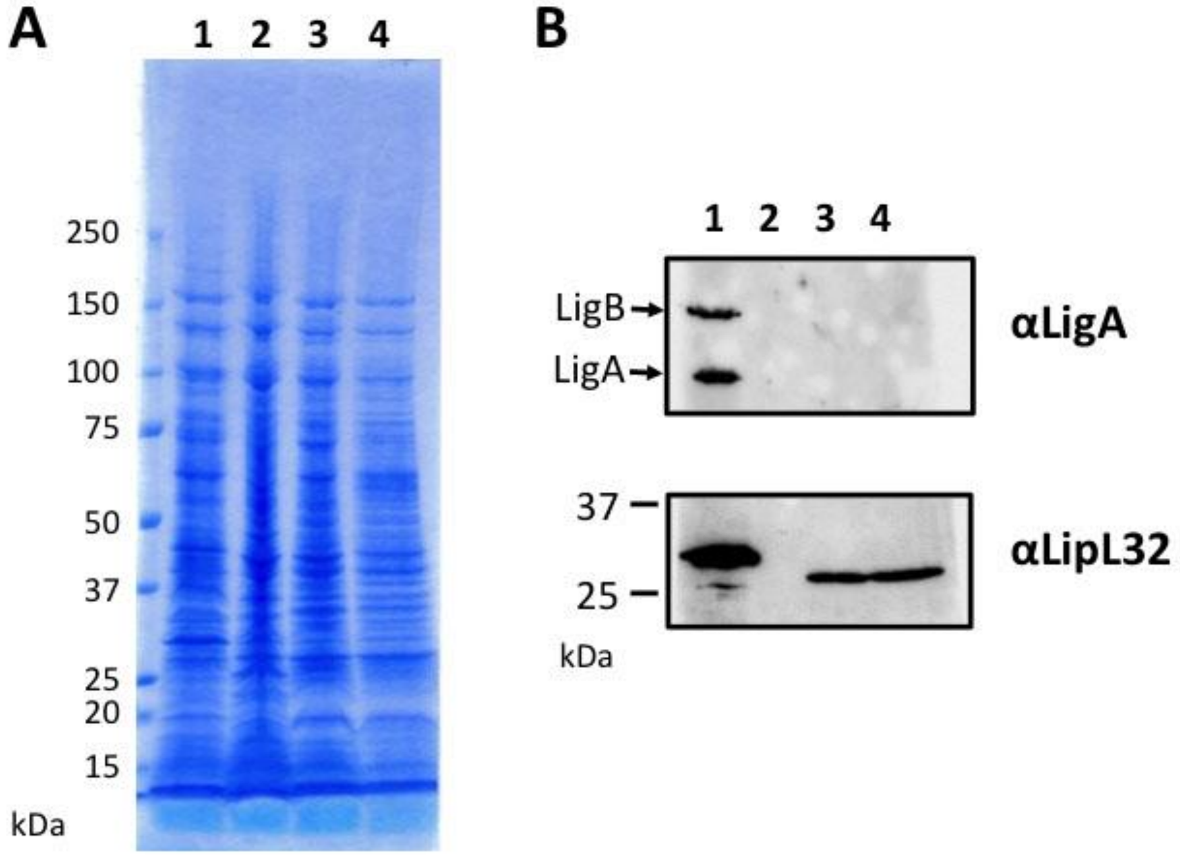

Technical Appendix Figure 2. Biological characteristics of *Leptospira* isolate NIID18 obtained from patient with *Leptospira licerasiae* infection. In the presence of 8-azaguanine in EMJH medium, NIID18, *L. interrogans* serovar Manilae strain UP-MMC-NIID and *L. licerasiae* strain VAR 010^T failed to grow, whereas *L. biflexa* serovar Patoc strain Patoc I^T grew well. About 5 × 10^8 leptospiral cells were suspended in 40 μL of 1× SDS-PAGE sample buffer. Fifteen microliters of each sample was subjected to 4%–20% SDS-PAGE (A) and Western blotting with anti-LigA (B, upper panel) or anti-LipL32 (B, lower panel) was performed on *Leptospira* whole cell lysates. As in the previous study (*1*), LigA nor B was not expressed in NIID18, but LipL32 was detected in NIID18 at the same molecular mass as that in VAR 010. Lane 1, *L. interrogans* serovar Manilae strain UP-MMC-NIID; lane 2, *L. biflexa* serovar Patoc strain Patoc I^T; lane 3, NIID18; lane 4, *L. licerasiae* strain VAR 010^T.

Reference

1. Matthias MA, Ricaldi JN, Cespedes M, Diaz MM, Galloway RL, Saito M, et al. Human leptospirosis caused by a new, antigenically unique *Leptospira* associated with a *Rattus* species reservoir in the Peruvian Amazon. PLoS Negl Trop Dis. 2008;2:e213. PubMed http://dx.doi.org/10.1371/journal.pntd.0000213
